# Research on the Stress Characteristics of Reuse of Semi-Rigid Base

**DOI:** 10.3390/s24248004

**Published:** 2024-12-14

**Authors:** Liting Yu, Dong Tang, Haoyi Kang, Haiqi He, Donliang Hu, Rui Li, Jianzhong Pei, Shihui Cheng

**Affiliations:** 1 Xinjiang Key Laboratory of Green Construction and Smart Traffic Control of Transportation Infrastructure, Xinjiang University, Urumqi 830017, China; 2 School of Traffic and Transportation·Engineering, Xinjiang University, Urumqi 830017, China; 3 School of Highway, Chang’an University, Middle Section of South Erhuan Road, Xi’an 710064, China

**Keywords:** semi-rigid base, pavement maintenance, stress characteristics, finite element, radar

## Abstract

Semi-rigid bases are widely used in road construction due to their excellent properties, high rigidity, and frost resistance, and they have been in service for many years. However, as the service life increases, the maintenance demands also grow, with traditional maintenance methods still being the primary approach. Based on a typical case using ground-penetrating radar (GPR) technology, this study explores the issue of cracks in semi-rigid bases and their impact on overlay layers. The findings indicate that the overlay layer at semi-rigid base cracks struggles to withstand significant tensile and shear stresses, leading to reflective cracking and reducing pavement durability. To address this problem, this paper investigates the application potential of crushing technology in maintaining semi-rigid bases. Crushing technology has been widely employed in the maintenance of cement concrete panels, effectively eliminating reflective cracks and extending the service life of overlays. Given that semi-rigid bases share similar high-strength characteristics with cement concrete panels, crushing technology shows considerable applicability in semi-rigid base maintenance. This study employs a finite element analysis method to establish a semi-rigid base model under the impact load of multi-hammer equipment. It examines its dynamic mechanical response and evaluates the feasibility and effectiveness of crushing technology for semi-rigid base maintenance. Additionally, this study investigates the influence of the crushed layer’s modulus and thickness on key mechanical design indicators of the overlay and proposes recommendations for optimal design parameters. The research results provide valuable references for the design of the thickness and modulus in maintaining and repairing semi-rigid bases, contributing to improving pavement performance and durability.

## 1. Introduction

Semi-rigid bases are widely used in asphalt pavements due to their high stiffness, excellent frost resistance, and use of low-cost materials such as cement, crushed stone, fly ash, and sand. These materials are readily available locally, making semi-rigid bases an economical and practical solution [1,2,3]. However, as pavement service life increases, the issue of cracking in semi-rigid bases become more prominent. These cracks gradually expand under traffic loads and temperature variations, propagating upwards to the surface layer and forming reflective cracks [4,5]. Reflective cracks reduce the durability of pavements and allow water infiltration and surface mud issues, further weakening the pavement’s bearing capacity and shortening its service life [6].

Cracking techniques have been extensively studied and applied internationally. In the United States, these techniques have been used in over 40 states to address large-scale cement concrete pavement damage [7]. Research by the University of Michigan emphasized the importance of pre-treatment, such as milling the asphalt layer, to ensure structural integrity. It is recommended that concrete particle sizes be reduced to below six inches to optimize performance. On Colorado’s I-76 highway, studies using multi-hammer breaking (MHB) and resonant breaker equipment demonstrated the effectiveness of cracking techniques in enhancing overlay durability. Japanese researchers have primarily investigated the mechanisms of reflective crack propagation under different loading conditions, providing references for optimizing pavement overlay design [8,9]. German research has focused on maximizing the performance of cracked layers and developing advanced overlay materials to minimize reflective cracks [10]. Additionally, British studies highlighted the influence of the cracked layer modulus and thickness on pavement structural performance, offering critical parameters for pavement maintenance design [11]. Scholars have concentrated on applying cracking techniques in cement concrete and semi-rigid bases in China. Studies have shown that the dynamic performance of cracked layers directly affects the long-term durability of pavements, and reasonable cracked layer parameter design is critical to ensuring maintenance effectiveness [12,13,14]. Similarly, Australian research has focused on extending pavement service life through cracking techniques and has proposed optimization recommendations for cracking equipment [15].

Despite significant theoretical and practical achievements, specific studies on applying cracking techniques in semi-rigid bases remain insufficient. Existing research has primarily focused on cement concrete pavements, with limited investigation into the mechanical behavior of semi-rigid bases after cracking [16,17,18]. For example, the mechanisms by which the cracked layer modulus and thickness affect the overlay performance need further clarification, as these factors are crucial for optimizing the design parameters [19].

This study addresses the above research gaps by exploring the potential applications of cracking techniques in semi-rigid base maintenance. Using finite element modeling, it analyzes the dynamic mechanical response of semi-rigid bases under multi-hammer impact loads. It focuses on the effects of the cracked layer modulus and thickness on the key mechanical design parameters of overlays [20,21]. Additionally, this paper provides parameter recommendations for optimizing cracked layer design to enhance the effectiveness of cracking techniques in semi-rigid bases [22,23,24]. Future research will further optimize cracking equipment, improve construction efficiency, and explore integrating cracking techniques with other maintenance methods to develop new materials, achieving more sustainable pavement management [25,26].

## 2. Methodology

### 2.1. Typical Detection by Ground-Penetrating Radar

**Figure 1:**
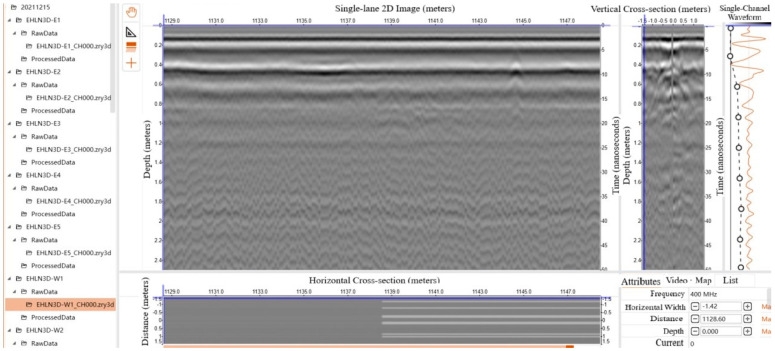
Typical detection of pavement.

As can be seen in Figure 1, there is an anomaly in the position of about 1 m below the ground, and the radar wave energy in the region where the target body exists is perturbed, with the waveform forming a “hyperbolic” shape and a strong reflection energy perturbation. The rightmost vertical line shows the single-channel waveform change in the cross section.

Through considerable depth, ground-penetrating radar can effectively predict the underground fracture zone’s existence area, scale, and influence range. Through enormous depth, ground-penetrating radar can effectively detect the location of anomalies within 100 m of the ground and, combined with a lower-frequency antenna, can simultaneously realize the detection of a greater depth of the target body. The latest ground-penetrating radar system effectively solves the attenuation problem of high-frequency electromagnetic waves. It dramatically expands the limited detection range while considering the advantages of traditional radar, thus realizing the detection in more application scenarios and solving many geologic problems that could not be solved before. It can be widely used in the detection of urban underground space.

These cases show the effectiveness and practicability of ground-penetrating radar technology in road detection, especially in detecting underground anomalies such as road cavities, underground dark river caverns, and underground rocks. Ground-penetrating radar technology provides a fast, accurate, and non-destructive solution.

### 2.2. Strength Formation Mechanism

**Figure 2:**
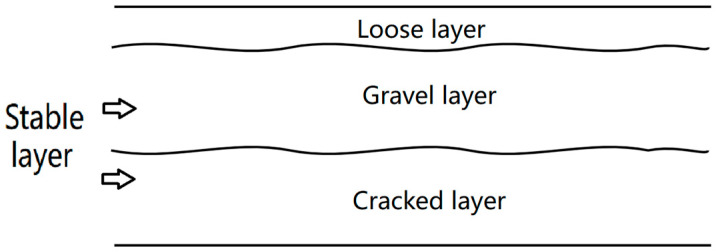
The stratum of the semi-rigid base layer after petrification.

This study categorizes the structure into different layers based on the particle size: a loose layer, a gravel layer, and a cracked layer. The composition of each layer, corresponding to varying degrees of crushing strength, is qualitatively analyzed, as illustrated in Figure 2.

**Figure 3:**
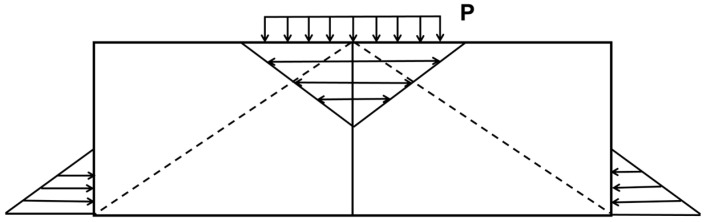
Generation of horizontal squeezing force.

The loose layer had no cement, the lowest strength of the three structural layers. Proper adjustment of the parameters of the equipment could reduce the excess crushing work and the thickness of the loose layer while ensuring that the semi-rigid base layer is completely crushed. The structural strength of the loose layer is too low to meet the structural strength requirements. The point distribution of the falling hammers of the multi-hammer head crushing plant is uneven, and the thickness of the loose layer is not uniform in the direction of travel. Therefore, the thickness of the loose layer should be as thin as possible to reduce the excess crushing work. The small thickness of the loose layer and the low modulus are neglected in the subsequent simulations. The shear strength between the cracked layer blocks mainly comprises the squeezing capacity between the blocks and the “arch effect” [27], as shown in Figure 3.

### 2.3. Mechanical Characteristics of Asphalt Overlay Under Different Crushing Effects

In the crushed layer, the degree of crushing is reflected in the size and distribution of the ground particles. A small broken particle size could better delay the occurrence of reflective cracks in the overlay, while a particle size that is too small would result in an overly thick overlay. A large crushing particle size has a poor effect on preventing and delaying the occurrence of reflective cracks in the overlay. In addition, due to the higher residual strength, the thickness of the overlay could be reduced. Therefore, the stress characteristics of the overlay layer are analyzed from the four aspects of the thickness and modulus of the gravel layer and the cracked layer. The stress characteristics of the overlay layer under different crushing degrees are discussed, and the best thickness and modulus combination of the gravel layer and the cracked layer are determined.

### 2.4. Finite Element Model

#### 2.4.1. Pavement Structure Model and Parameters

**Figure 4:**
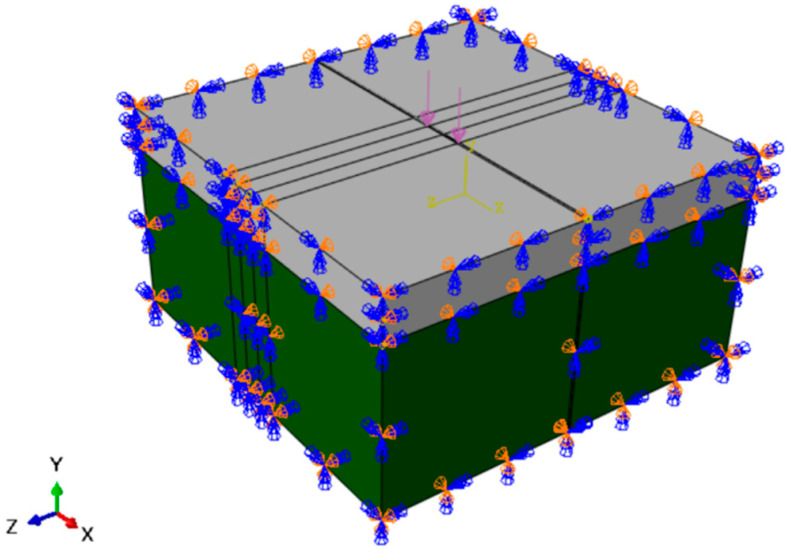
Pavement structure model diagram.

The model adopts the dynamic analysis method. The pavement structure is composed of both a semi-rigid base and soil. The purple arrows in the center of the model are loads and the dark blue arrows around them are constraints. The geometric dimensions of the model are shown in Figure 4 below.

The pavement structure is divided into the base layer and the subgrade (fixed boundaries). The base layer has a modulus of 1500 MPa and a thickness of 40 cm, while the subgrade has a modulus of 60 MPa and a thickness of 2 m. The structural materials are assumed to be elastic, homogeneous, and isotropic. The contact between layers is continuous in all directions, and the influence of gravity on each structural layer of the pavement is considered negligible.

#### 2.4.2. MHB Drop Hammer Load and Acting Position

The MHB (multiple-head break) equipment used in this study is crushing equipment with multiple heavy hammers. This kind of equipment hits the concrete slab through the fall of a heavy hammer, thereby breaking the concrete slab [28]. This study uses high-amplitude, low-frequency drop hammers to fracture the semi-rigid base panel, facilitating related research and analysis.

The MHB drop weight impact model applies transient loads, simulating where a drop weight falls from a specified height and impacts the semi-rigid base at a certain velocity, resulting in potential fracturing [29,30]. The parameters of the MHB equipment are detailed in Table 1.

**Figure 5:**
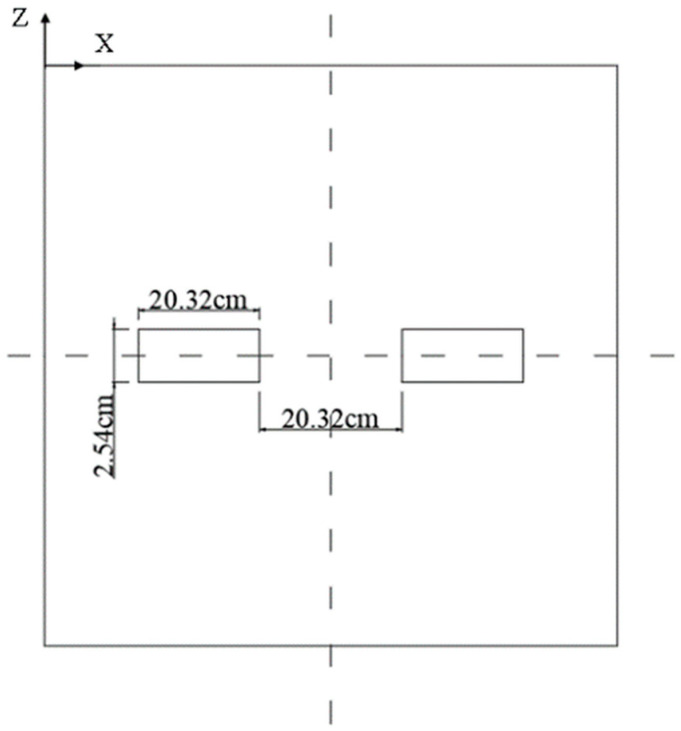
Schematic diagram of impact load acting position.

The load acted as shown in Figure 5 below.

In this paper, the transient impact load is used to simulate the drop weight load, so it is necessary to determine the impact load when the drop weight falls on the surface of the semi-rigid base [31,32]. The impulse is used to calculate the impact load inversely, and the formula is as follows:

(1) F=mv0−mvtt

Among them, F—impact force (N);

m—MHB single-hammer weight (kg);

t—duration of impact (s);

$v_{0}$, $v_{t}$—MHB hammer ground velocity before and after the action of the heavy hammer (m/s).

After consulting and referring to the MHB series calibration parameters, it could be known that the load is maintained at the peak value for 60 ms after the drop hammer touches the semi-rigid base. Then, the load is reduced to 0 after 200 ms, and the drop hammer height is 1.25 m. The grounding area of the falling weight is $A=\left(20.32+2.54\right)\;{cm}^{2}$, and the reason is that the contact stress shows a triangular distribution of first increasing and then decreasing. The maximum peak stress is obtained from the following equation:

(2) $$P_{max}=2\bar{P}$$

(3) P¯=FA

**Figure 6:**
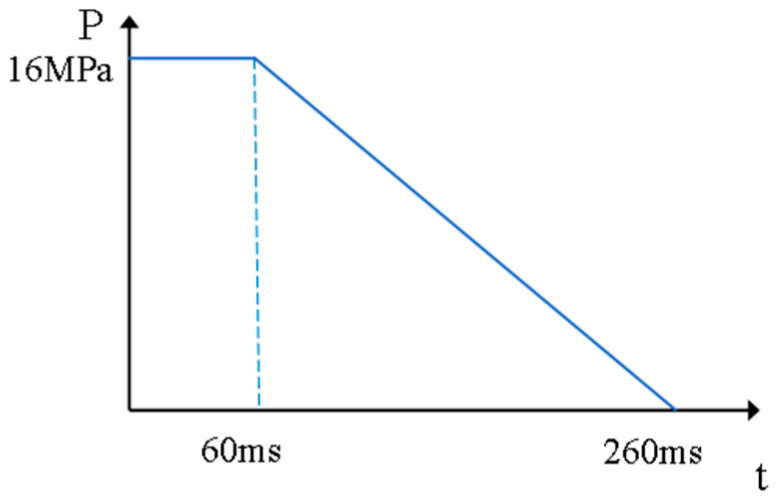
Diagram of impact load action.

The result is $P_{max}=16$ MPa. The schematic diagram of the MHB single-impact load is shown in Figure 6 as follows:

#### 2.4.3. Stiffness Element Distribution

**Figure 7:**
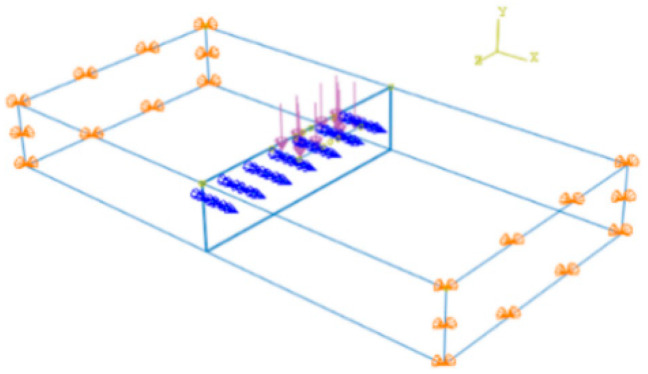
Distribution of rigid elements.

**Figure 8:**
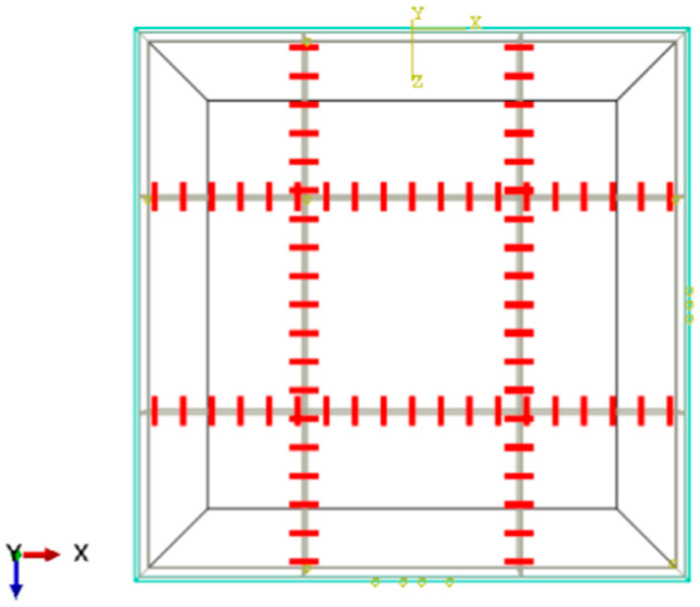
Rigid element model of pavement structure.

A rigid element is set in the middle of the plates to simulate the shear strength between the blocks, and the load transfer coefficient is used to represent the shear strength between the plates. Figure 7 and Figure 8 show that the rigid elements are 20 cm long and evenly arranged with a spacing of 20 cm, the middle part of the load-acting plate (As indicated by the red line).

The model adopts the Winkler foundation model and uses the joint load transfer capacity to characterize the shear strength between the blocks after the semi-rigid base is microcracked [31,33]. The load transfer capacity could be described by the load and stress of the loaded and unloaded plates on both sides of the joint and by indicators such as the strain and deflection. This study uses deflection to describe, as shown in the following formula:

(4) $$LTE=\frac{w_{U}}{w_{L}}\times 100\%$$

In the formula, LTE—load transfer capacity between blocks;

$w_{U}$—the maximum vertical displacement of the unloaded plate; $w_{L}$—the maximum vertical displacement of the loaded plate.

## 3. Results of the Research

### 3.1. Stress Distribution in All Directions

The origin is set at the center of the board, X and Y are the width and depth direction of the road, and Z is the driving direction. In this paper, we discuss the stress distribution in all directions in the semi-rigid base under the impact of the drop hammer.

**Figure 9:**
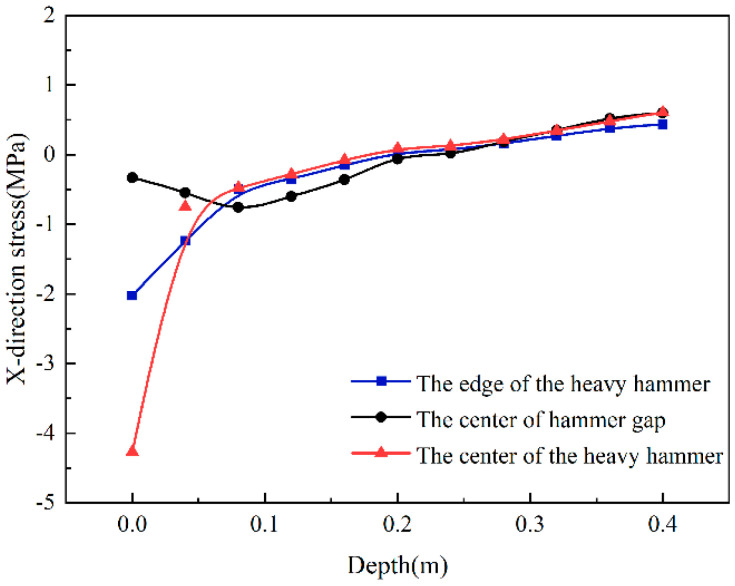
X-direction stress changes with depth.

It can be seen from Figure 9 that along the depth direction of the road surface, the stress change trend in the X-direction at the center and edge of the weight is consistent. In the upper part of the semi-rigid base, the center and the edge of the weight are in a high-pressure stress state, and as the depth increases, the compressive stress gradually decreases. When the center of the weight is 22 cm in depth, the stress state changes from compressive to tensile stress. The edge of the heavy hammer changes from compressive to tensile stress at a depth of 18 cm. The result shows that the force in the X-direction is similar between the weight’s center and edge. The force state of the center in the hammer gap differs from the previous two. At the top of the semi-rigid base, the center compressive stress of the hammer gap is 0.33 MPa. As the depth increases, the compressive stress first increases and then decreases, and the compressive stress changes to tensile stress at a depth of 19 cm. The reason is that the load does not directly act on the center of the hammer gap, where the compressive stress is minor. As the depth increases, the stress state of the center in the hammer gap is affected by the high-pressure stress at the center of the heavy hammer; in this case, the compressive stress becomes larger. When the depth is greater than 10 cm, the changing trend in the force state along the depth direction of the center in the hammer gap is consistent with the center of the heavy hammer.

**Figure 10:**
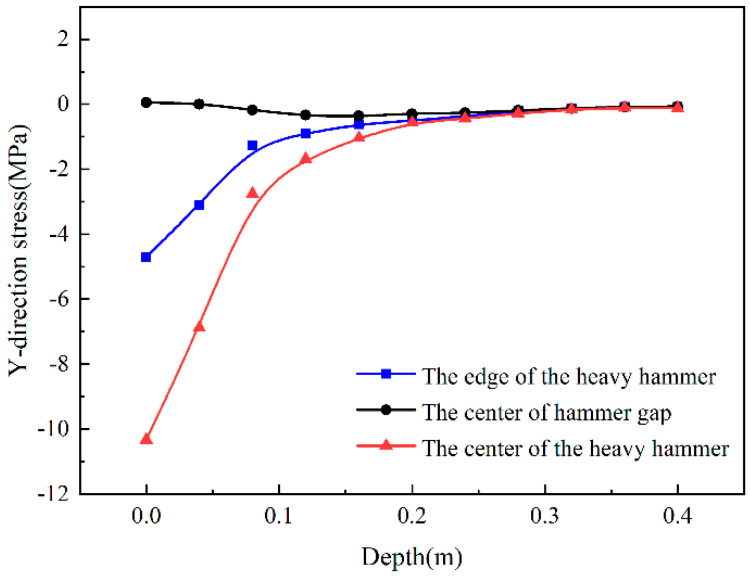
Y-direction stress changes with depth.

Figure 10 is a trend diagram of the Y-direction stress changing with the depth direction (i.e., from the road surface to the subgrade). It compares the center and the edge of the heavy hammer. The stress value at the center of the hammer gap is relatively small, which shows a trend of first increasing and then decreasing. The center and the edge of the heavy hammer are still in a state of high-pressure stress on the top of the semi-rigid base. The stress in the hammer’s center is 52% higher than the edge of the hammer. After a depth of 28 cm, the Y-direction stress at the three positions tends to be the same, approaching zero. The results showed that the vertical stress at the hammer’s center and the hammer’s edge is mainly distributed in part above 28 cm, and the vertical stress at the center of the hammer gap is relatively small.

**Figure 11:**
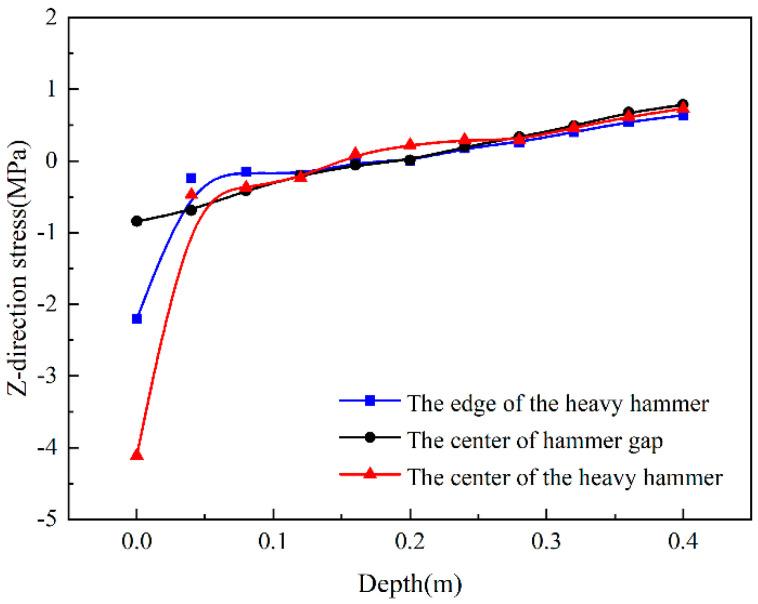
Z-direction stress changes with depth.

Figure 11 is a trend diagram of the Z-direction stress changing with the depth direction, which can be seen from the figure that compares the center of the heavy hammer and the edge of the heavy hammer, while the stress value at the center of the hammer gap is relatively small, which shows a trend of first increasing and then decreasing. The figure shows that the trend diagram of the Z-direction stress changes with the depth direction, which could be seen as the stress trends along the driving direction at the three locations are the same. After a depth of 28 cm, the stress in the driving direction tends to be the same in the three positions.

**Figure 12:**
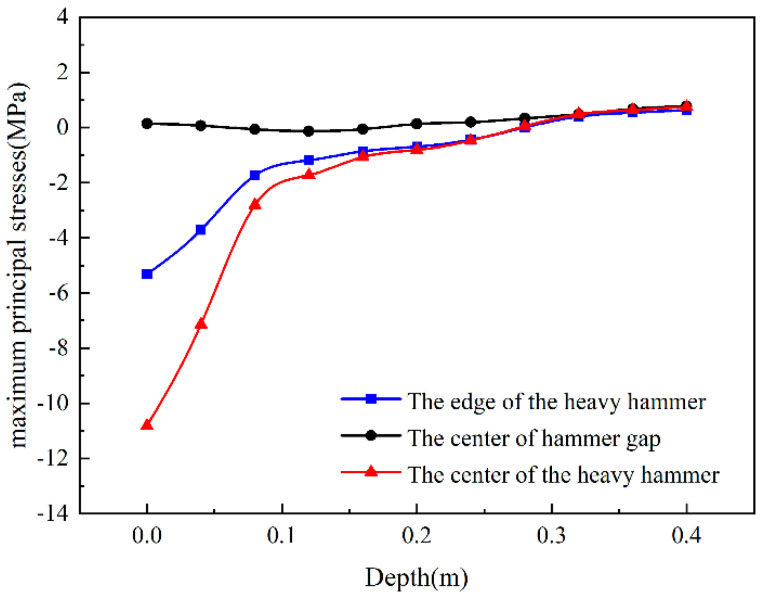
The maximum principal stress varies with depth.

Figure 12 shows that the maximum principal stresses at the three locations are all compressive. The maximum principal compressive stress on the center and edge of the hammer is relatively large, and the maximum principal stress at the hammer’s center is 48% of the edge of the hammer. After the hammer’s center is 18 cm, the maximum principal stress transforms into tensile stress. In addition, the maximum is 0.7 MPa at the bottom of the semi-rigid base slab, and the splitting strength of the cement-stabilized gravel semi-rigid base is 0.6 MPa. The results show that the bottom of the semi-rigid base plate begins to fracture after the drop hammer action, and the multi-hammer head crushing equipment is suitable for the semi-rigid base.

**Figure 13:**
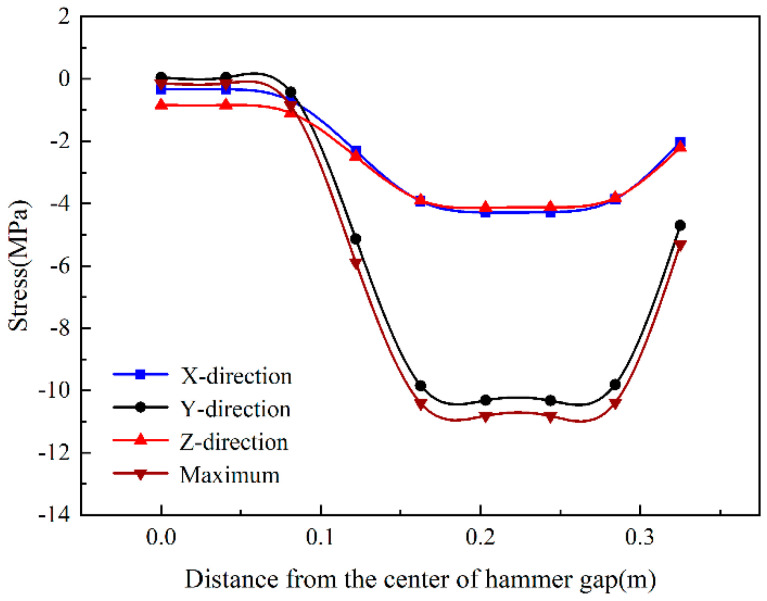
The stress in each direction of the surface changes with the X-direction.

Figure 13 shows the trend of the stress in each direction along the X-direction. The results show that the four stresses have the same changing trend along the X-direction. The stress at the center of the hammer gap is the lowest, and the closer the weight is applied, the greater the stress reaches its maximum value at the center of the weight. The X-direction and Z-direction stress are the same, and the Y-direction stress and its changing law are very close to the maximum principal stress. The results show that the Y-direction dominates the maximum principal stress direction.

### 3.2. Analysis of Strength Attenuation of Pavement Structure

After the semi-rigid base is crushed, the cracks formed in the base are not vertical and smooth, which moving upwards diagonally, and the contact surface is rough and uneven. Therefore, the frictional part of the contact surface provided by the inclined and uneven surface forms the vertical shear strength between the blocks.

**Figure 14:**
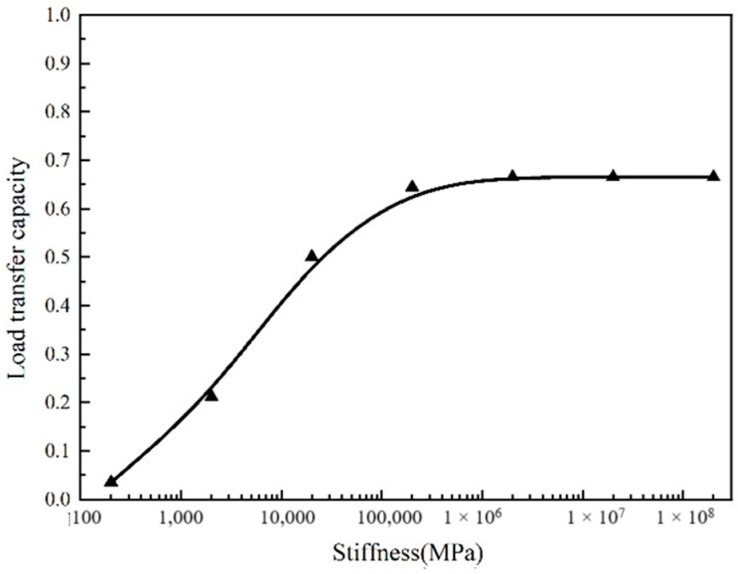
Load transfer capacity changes with stiffness.

Figure 14 shows the changing trend in the load transfer capacity between blocks with stiffness. From the figure, it can be seen that when the rigid units are uniformly arranged at a distance of 20 cm, the load transfer capacity remains stable after the stiffness of the rigid units reaches 200 Gpa. In this paper, when the load transfer capacity is 0.2, the block is defined as a weak inter-lock. When the load transfer capacity is 0.4, it is described as a medium inter-lock; when the load transfer capacity is 0.6, it is a strong inter-lock. By the interpolation method, it could be calculated that when the rigid unit modulus is 2000 MPa, it is a weak inter-lock, 9000 Mpa is a medium inter-lock, and 100 Gpa is a strong inter-lock.

**Figure 15:**
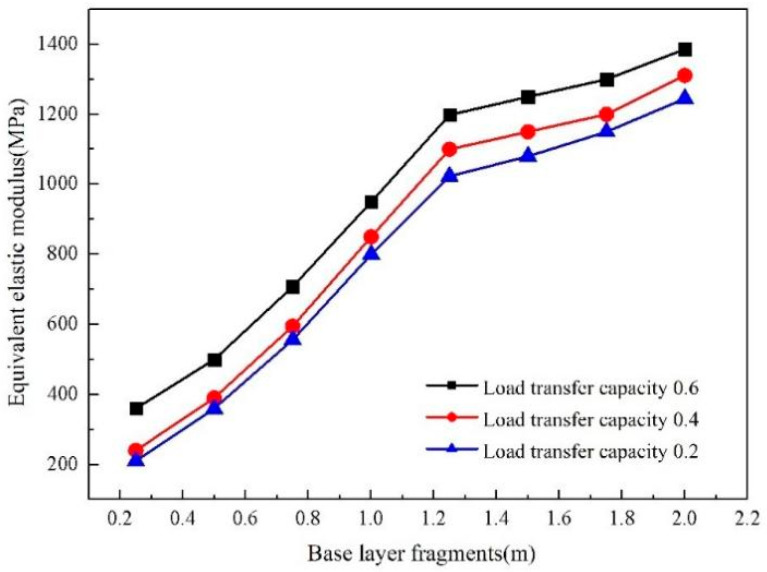
The change in equivalent elastic modulus with different base fragments.

The modulus of the crushing semi-rigid base layer strength is reduced by adopting the equivalent method of pavement bearing. The semi-rigid base layer modulus after petrification is called the equivalent elastic modulus. The pavement structure model is shown in Figure 15. The short red lines represent rigid elements. Figure 15 shows that when a fragment is more excellent than 1.25 m, the modulus decreases slowly, and the elastic modulus decreases faster when less than 1.25 m.

## 4. Discussion and Interpretation of Results Obtained

### 4.1. The Effect of Modulus Changes on Overlay

When the crushed particle size is too small, and the equivalent modulus is too low, the thickness of the overlay layer increases. Combined with the results of crushing cement concrete slabs, it could be considered that the modulus of the crushed rock layer should be greater than 200 MPa, and the particle size of the cracked layer should be more significant than that of the crushed rock layer. Therefore, the modulus of the cracked layer is 200 MPa to 1500 MPa, and the increment is 200 MPa to calculate the force characteristics of the asphalt overlay.

**Figure 16:**
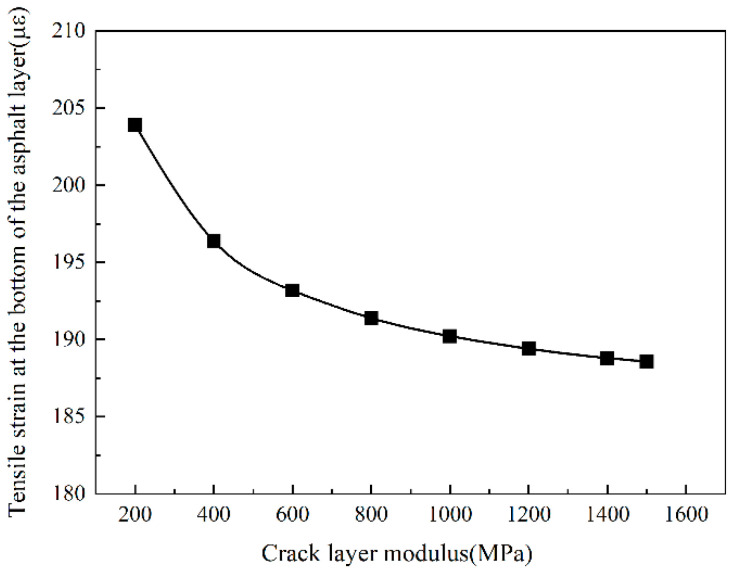
The tensile strain of the bottom of the asphalt layer.

It can be seen from Figure 16 that the tensile strain at the bottom of the asphalt layer gradually decreased as the modulus of the cracked layer increased. When the modulus of the cracked layer is less than 1000 MPa, the tensile strain of the bottom of the asphalt layer decreases rapidly. When the modulus of the cracked layer is more remarkable than 1000 MPa, the tensile strain of the bottom of the asphalt layer decreases slowly. In general, when the modulus of the cracked layer increases from 200 MPa to 1500 MPa, the tensile strain decreases from 204 με to 189 με, a total decrease of 6.25%, which shows that the tensile strain of the bottom layer of the asphalt layer decreases with the increase in the modulus of the cracked layer. However, the reduction is not significant, so it could be considered that the tensile strain has little relationship with the modulus of the cracked layer.

**Figure 17:**
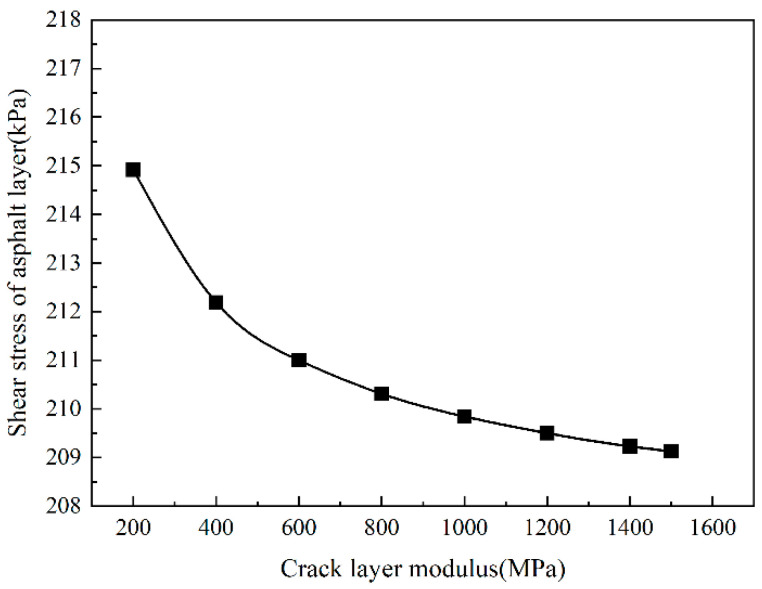
The shear stress of the asphalt layer change with crack layer modulus.

Figure 17 shows that the shear stress of the asphalt layer gradually decreases as the modulus of the cracked layer increases. When the modulus of the cracked layer is less than 1000 MPa, the shear stress of the asphalt layer decreases rapidly, and when the modulus of the cracked layer is more significant than 1000 MPa, the shear stress of the asphalt layer decreases slowly. In general, when the modulus of the cracked layer increases from 200 MPa to 1500 MPa, the shear stress of the asphalt layer decreases by 5.9 kPa, which indicates that the shear stress of the asphalt layer decreases with the increase in the modulus of the cracked layer.

**Figure 18:**
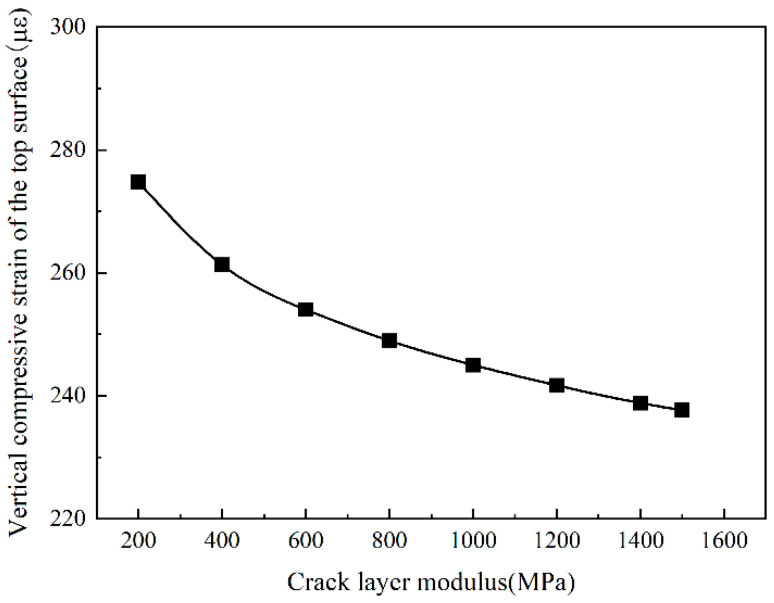
The vertical compressive strain of the top surface of the subgrade Change with Crack Layer Modulus.

**Figure 19:**
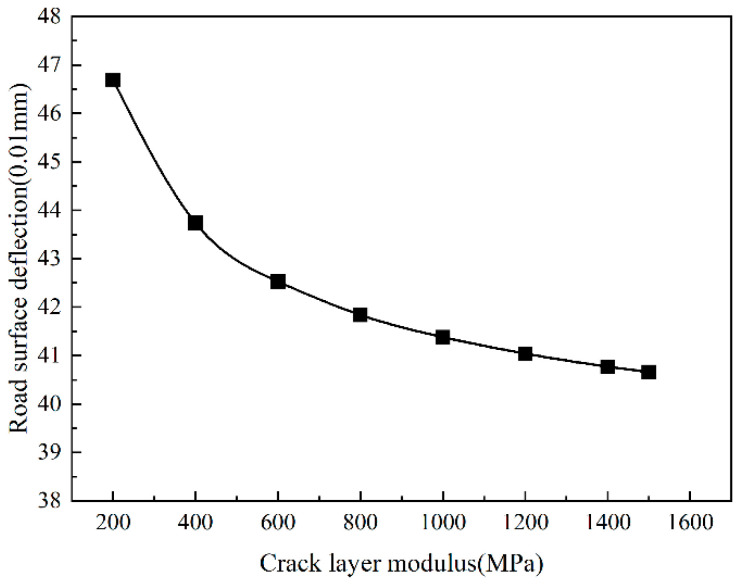
Road surface deflection change with crack layer modulus.

It can be seen from Figure 18 and Figure 19 that the trends in the road surface deflection and subgrade top surface compressive strain with the modulus of the cracked layer are consistent with the tensile strain at the bottom of the asphalt layer. As the modulus of the cracked layer increases, the road surface deflection and the vertical compressive strain of the top surface of the roadbed gradually decrease. When the modulus of the cracked layer is less than 1000 MPa, the road surface deflection and the vertical compressive strain of the top surface of the roadbed decrease rapidly. When the modulus of the cracked layer is more significant than 1000 MPa, the road surface deflection and the vertical compressive strain of the roadbed top surface slowly decrease. Overall, the reduction is not essential.

In summary, increasing the modulus of the cracked layer could reduce the shear stress of the asphalt layer, the tensile strain at the bottom of the layer, the vertical compressive strain of the top surface of the subgrade, and the surface deflection to a certain extent. When the modulus of the cracked layer is lower than 1000 MPa, the rate of decrease is faster, and the amplitude is more significant. When it is greater than 1000 MPa, the rate of decline is slow. Excessive modulus of the cracked layer would affect the effect of preventing reflection cracks on the asphalt layer. Therefore, the modulus of the cracked layer should not be too high, and the modulus of the cracked layer should be taken as 1000 Mpa.

The modulus of the gravel layer ranges from 200 MPa to 1000 MPa, with an increment of 200 MPa.

**Figure 20:**
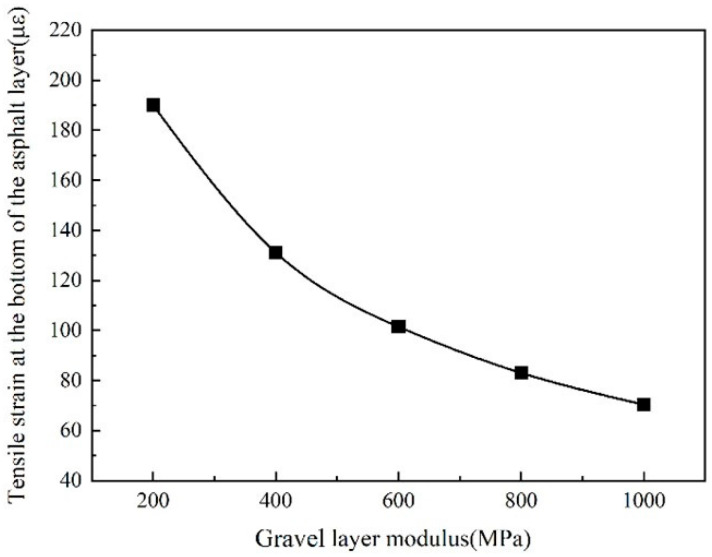
The tensile strain at the bottom of the asphalt layer change with gravel layer modulus.

It can be seen from Figure 20 that the tensile strain at the bottom of the asphalt layer gradually decreased as the modulus of the gravel layer increased. The rate of decrease in the tensile strain at the bottom of the asphalt layer decreases with the increase in the modulus of the gravel layer. When the modulus of the gravel layer increased from 200 MPa to 400 MPa, the tensile strain at the bottom of the asphalt layer was reduced by 30.9% (58.9 με). When the modulus of the gravel layer increased from 400 MPa to 1000 MPa, the tensile strain at the bottom of the asphalt layer decreased by 32.1% (60.1 με), indicating that the modulus of the gravel layer has a more significant influence on the tensile strain at the bottom of the asphalt layer.

**Figure 21:**
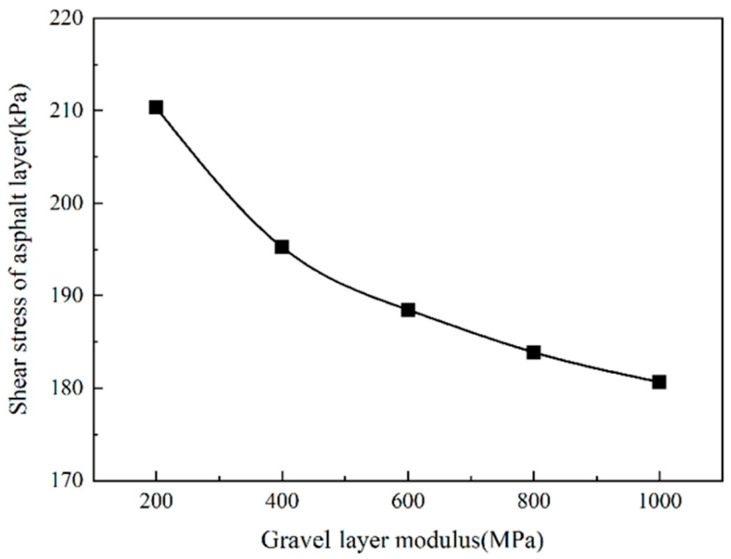
The shear stress of the asphalt layer changes with the gravel layer modulus.

Figure 21 shows that the shear stress of the asphalt layer gradually decreases as the modulus of the gravel layer increases. When the modulus of the gravel layer is less than 400 MPa, the shear stress of the asphalt layer decreases rapidly. When the modulus of the gravel layer is more remarkable than 400 MPa, the shear stress of the asphalt layer decreases slowly. When the modulus of the gravel layer increases from 200 MPa to 400 MPa, the shear stress of the asphalt layer is reduced by 6.9% (14.61 kPa). When the modulus of the gravel layer increases from 400 MPa to 1000 MPa, the shear stress of the asphalt layer is reduced by 7.4% (14.2 kPa). The results show that the modulus of the gravel layer has a specific influence on the shear stress of the asphalt layer.

**Figure 22:**
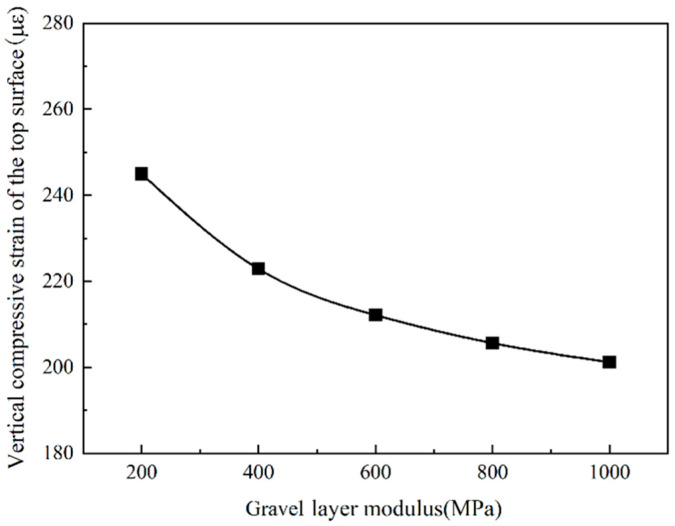
The vertical compressive strain of the top surface of the subgrade.

**Figure 23:**
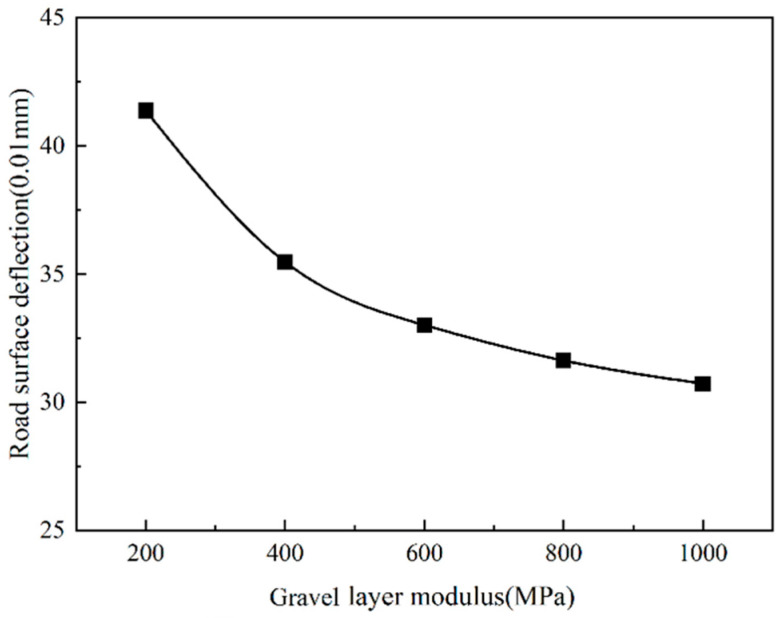
Road surface deflection change with gravel layer modulus.

It can be seen from Figure 22 and Figure 23 that the road surface deflection and the compressive strain of the top surface of the roadbed are the same as the tensile strain at the bottom of the asphalt layer, which changes with the change in the modulus of the gravel layer. As the modulus of the cracked layer increases, the road surface deflection and the vertical compressive strain of the top surface of the roadbed gradually decrease. When the modulus of the gravel layer is less than 400 MPa, the road surface deflection and the vertical compressive strain of the top surface of the roadbed decrease rapidly. When the modulus of the cracked layer is more remarkable than 400 MPa, the road surface deflection and the vertical compressive strain of the top surface of the roadbed slowly decrease.

Due to the larger crushed particle size, the mechanical indicators have decreased. However, the larger the particle size, the more likely reflection cracks will appear on the asphalt overlay. Therefore, the modulus of the gravel layer is controlled to 400 MPa, which not only maintains a specific structural strength but also effectively prevents the occurrence of reflection cracks.

### 4.2. The Influence of Thickness Change on Overlay

The crushed and cracked layers are, respectively, 4~36 cm, with an increase of 4 cm. The results show that the tensile strain at the bottom of the asphalt layer increased as the thickness of the gravel layer increased. When the thickness of the cracked layer increases from 4 cm to 36 cm, the tensile strain at the bottom of the asphalt layer rises by 79.9%. It can be seen from Figure 23 that when the thickness of the gravel layer is from 4 cm to 20 cm, the tensile strain growth rate is more prominent, a total increase of 71.6%. When the thickness of the cracked layer is changed from 20 cm to 36 cm, the tensile strain only increases by 9.2%.

**Figure 24:**
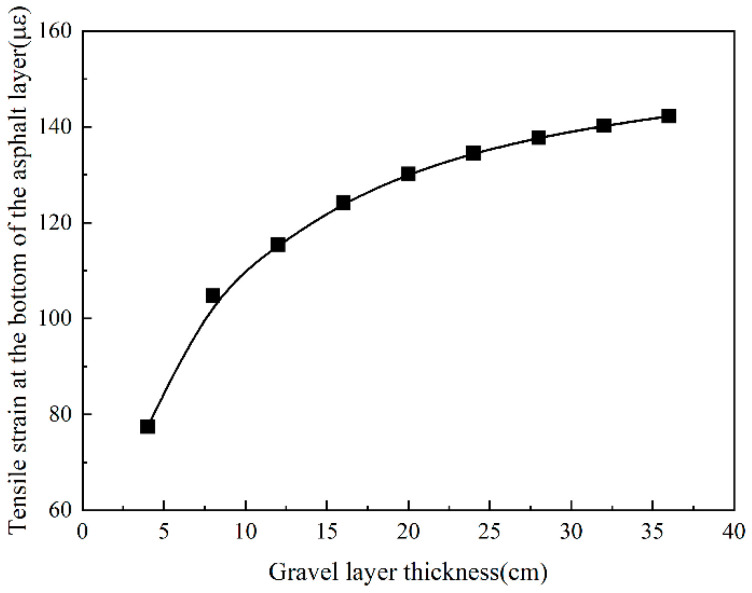
The tensile strain at the bottom of the asphalt layer changes with gravel layer thickness.

It can be seen in Figure 24 that when the thickness of the gravel layer is larger, the modulus of the original semi-rigid base layer decreases more; in this case, the integrity is poor. The additional asphalt layer is thicker, and the tensile strain of the asphalt layer is too significant. Therefore, reducing the thickness of the gravel layer is beneficial in lowering the tensile strain of the additional layer. As the thickness of the gravel layer is smaller, it indicates that the crushed particle size is too large. This would cause the effect of preventing reflection cracks to be less than expected; that is to say, the thickness of the gravel layer should not be too small. Compared with 4~20 cm, the tensile strain growth rate in the range of 20~36 cm is slower. Therefore, when the thickness of the gravel layer and the cracked layer are both controlled at 20 cm, not only could it effectively prevent reflection cracks, but it could also ensure that the tensile strain at the bottom of the asphalt layer would not be too significant.

**Figure 25:**
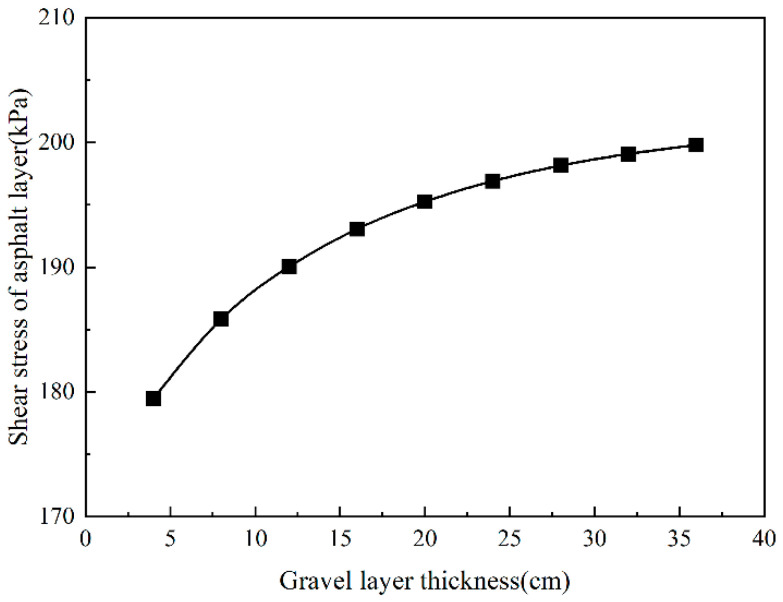
The shear stress of the asphalt layer changes with the gravel layer thickness.

It can be seen from Figure 25 that the changing trend in the shear stress of the asphalt layer is consistent with the tensile strain at the bottom of the asphalt layer. As the thickness of the gravel layer increased, when the thickness of the cracked layer increased from 4 cm to 36 cm, the shear stress of the asphalt layer increased by 11.5%. When the thickness of the gravel layer is less than 20 cm, the shear stress growth rate of the asphalt layer is more significant than that when the thickness of the gravel layer is greater than 20 cm. It shows that when the thickness of the gravel layer reaches 20 cm, the shear stress growth trend of the asphalt layer tends to be slow. Therefore, from the perspective of the shear stress of the asphalt layer, controlling the thickness of the gravel layer to 20 cm could effectively prevent reflection cracks and ensure that the shear stress of the asphalt layer is not too large.

**Figure 26:**
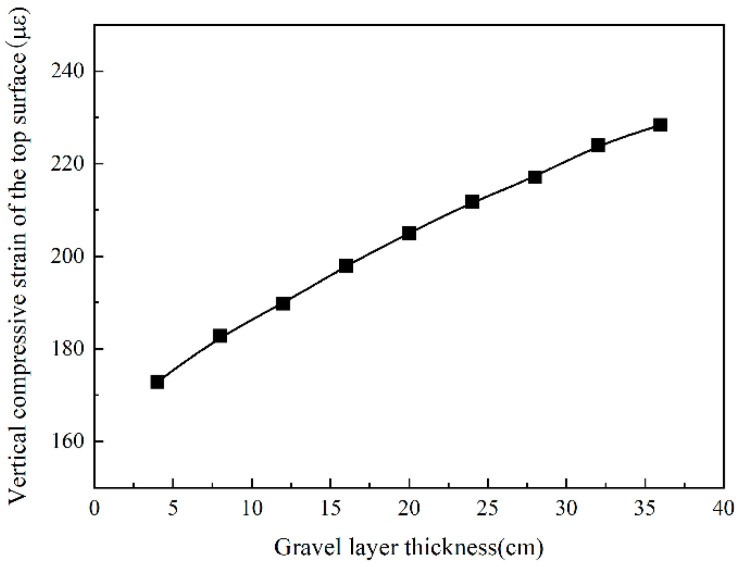
The vertical compressive strain of subgrade changes with gravel layer thickness.

From Figure 26, it can be seen that the vertical compressive strain on the top surface of the roadbed increases with the increase in the thickness of the gravel layer, which shows a linear growth trend. When the thickness of the gravel layer changes from 4 cm to 36 cm, the vertical compressive strain on the top surface of the roadbed increases by 38.5%. Therefore, the thickness of the gravel layer has a more significant impact on the compressive strain of the top surface of the subgrade.

**Figure 27:**
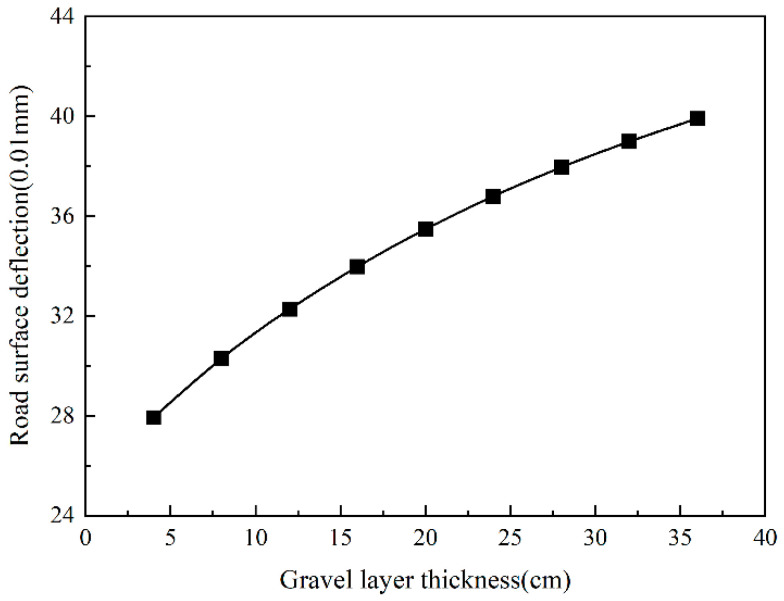
Road surface deflection changes with gravel layer thickness.

Combining Figure 26 and Figure 27, it could be seen that the trend in the road surface deflection with the thickness of the gravel layer is roughly similar to the vertical compressive strain of the top surface of the roadbed. However, when the thickness of the gravel layer is greater than 20 cm, the growth rate of the road surface deflection is reduced.

To sum up, to ensure the effective prevention of reflection cracks, the semi-rigid base layer should maintain a specific strength after being broken as much as possible, which could make the stress and strain of the asphalt layer, the vertical compressive strain of the top surface of the roadbed, and the road surface deflection small. In addition, it could be considered that the thickness of the gravel layer should be set to 20 cm.

### 4.3. Combination Structures

According to the different crushing effects of the crushed petrified layers, five structural combinations of crushed petrified layers are selected. The combinations are applied to the base and sub-base layers for ten structural combinations. The parameters of each structural combination are shown in Table 2, which contains a comparative analysis of the leading mechanical indicators of pavement structure design for different combinations of pavement structures under load action.

**Figure 28:**
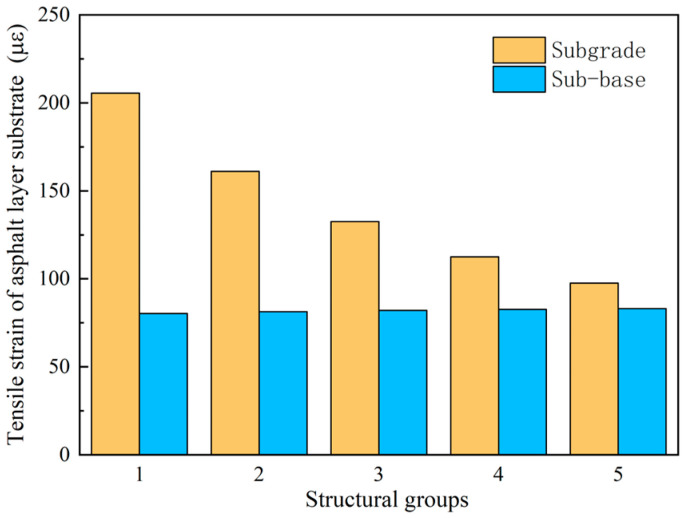
Tensile strain at the bottom of asphalt layer for different structural combinations.

As seen in Figure 28, when used as a roadbed, the higher the modulus of the crushed layer, the higher the modulus of the crushed layer. When changing from Structure 1 to Structure 5, the lower the tensile strain at the bottom of the asphalt layer, the lower the tensile strain at the base of the asphalt layer by 51.2% for different degrees of fragmentation. When the crushed petrochemical layer is used as a subgrade after adding a flexible base layer on top of the crushed petrochemical layer, the maximum difference in the tensile strain between the asphalt layers of the five structures is 8.2 μ. When the crushed petrochemical layer is used as the base and sub-base, respectively, the difference is 124.2 μ, 79.7 μ, 50.47 μ, 30.5 μ, and 14.5 μ for each structure combination.

**Figure 29:**
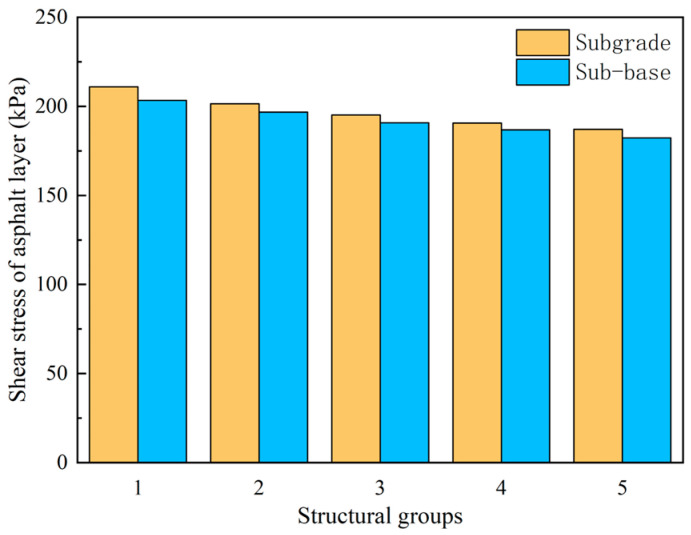
Shear stress in asphalt layers for different structural combinations.

Figure 29 shows that the more severely the semi-rigid subgrade is broken, the higher the shear stress in the asphalt layer, whether used as a subgrade or a base layer. The difference between the most severely broken Structure 1 and the least severely broken Structure 5 is 7.2 kPa when the crushed petrochemical layer is used as the subgrade and sub-base, respectively, while the difference is 4.3 kPa. The results indicate that the crushed petrochemical layer has less effect on the shear stress of the asphalt surface layer when used as a subgrade or base.

**Figure 30:**
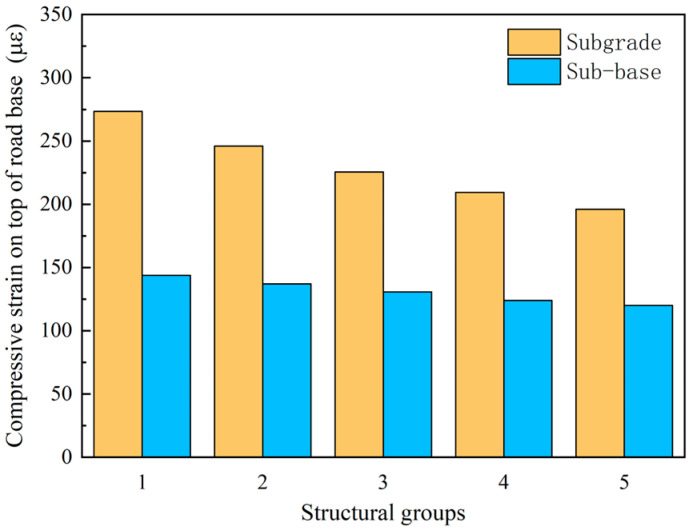
Compressive strain on top of road base for different structural groups.

The change in the vertical compressive strain at the top of the roadbed is consistent with the tensile strain at the base of the asphalt layer. As can be seen from Figure 30, when the crushed petrochemical layer is used directly as the subgrade, the top surface compressive strain is more incredible than 200 μ, and the top surface vertical compressive strain decreases by 27.4%. When the crushing degree is from Structure 1 to Structure 5, the top surface vertical compressive strain decreases by 13.5%. The results indicate that when directly used as a roadbed, the degree of fragmentation of the crushed layer significantly impacts the vertical compressive strain at the top of the roadbed.

**Figure 31:**
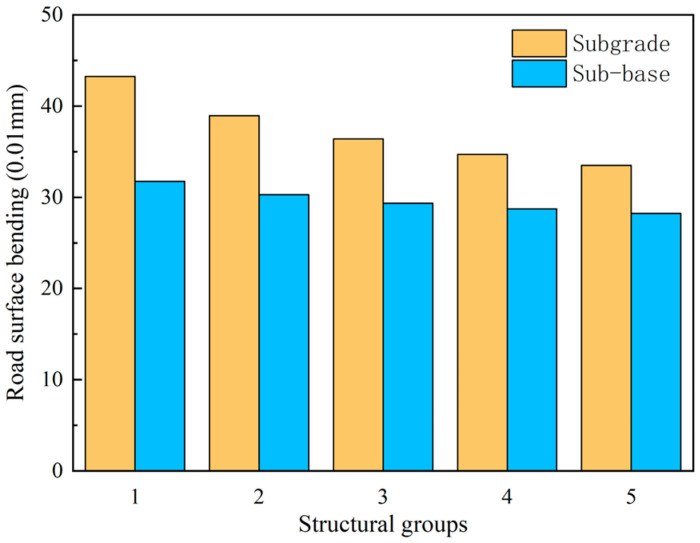
Surface bending settlement for different structural combinations.

As can be seen from Figure 31, the trends in the road surface bending are consistent with the above-mentioned tensile strains in the asphalt subgrade, shear stresses in the asphalt, and vertical compressive strains in the top surface of the road base, so they are not repeated here.

In summary, when the crushed petrified layer is used directly as the subgrade, the main mechanical indexes decrease significantly as the degree of crushing decreases. When the crushed petrified layer is used as a subgrade, the main mechanical indexes do not change much, mainly because adding the flexible base layer increases the overall load-bearing capacity of the pavement. The degree of crushing has less influence on the main mechanical indexes. From the perspective of preventing reflective cracks and increasing the load-bearing capacity of the pavement structure, regardless of the degree of crushing, when the crushed petrified layer is used as the base layer, the tensile strain at the bottom of the asphalt layer and the compressive strain at the top surface of the roadbed are too large. The bottom of the asphalt layer is prone to fatigue cracks. Therefore, the crushed petrochemical layer can only be used as a sub-base, which is also necessary to add a layer of sub-base on top of the crushed petrochemical layer to improve the overall bearing capacity of the pavement, reduce the tensile strain at the bottom of the asphalt layer and the compressive strain at the top of the roadbed, and achieve the effect of preventing reflection cracks.

## 5. Conclusions

1. After being crushed, the semi-rigid base layer is divided into three distinct layers: the loose layer, the gravel layer, and the cracked layer, each characterized by different mechanisms of shear strength. In the loose layer, shear strength is primarily governed by the material’s internal friction angle and the regular forces at the particle contact surfaces. For the gravel layer, the shear strength is mainly influenced by the internal friction angle and pre-compression stress. In contrast, the cracked layer’s shear strength is predominantly determined by the squeezing capacity between blocks and the arching effect.
2. The Abaqus finite element software was used to construct a multi-hammer crushing equipment model acting on a semi-rigid base pavement structure. The mechanical response of the semi-rigid base pavement under the impact load of the multi-hammer crushing equipment was analyzed. The results indicate that the maximum principal stress at the bottom of the semi-rigid base reaches 0.7 MPa, which exceeds its splitting strength of 0.6 MPa. Consequently, multi-hammer crushing equipment is adequate for crushing semi-rigid base pavement structures.
3. An analysis of the strength attenuation of the semi-rigid base layer after crushing reveals a clear relationship between the particle size and equivalent strength. As the particle size decreases, the equivalent strength of the base layer diminishes significantly. This trend highlights the particle size’s critical influence on the crushed layer’s structural integrity. Specifically, the decay in the equivalent strength becomes particularly pronounced when the particle size is reduced to less than 1.25 mm. At this threshold, the rapid degradation in the strength can compromise the load-bearing capacity of the layer, emphasizing the need for careful control and monitoring of the particle size during the crushing process to ensure the stability and functionality of the semi-rigid base.
4. An analysis of the asphalt overlay’s force characteristics under varying degrees of crushing revealed that the optimal design parameters include a gravel layer with a thickness of 20 cm and a modulus of 400 MPa and a crack layer with a thickness of 20 cm and a modulus of 1000 MPa. This configuration, particularly in its fractured state, effectively suppresses the formation of reflective cracks while maintaining the structural integrity and strength of the gravel and crack layers.
5. A comparison was conducted to analyze the changes in the structural mechanics of crushed petrified layers used as a subgrade and sub-base. The findings indicate that using the crushed petrified layer as the base layer results in excessive tensile strain at the bottom of the asphalt layer and compressive strain at the top of the road base. Consequently, utilizing the crushed petrified layer as a semi-rigid base layer is recommended. Additionally, a base layer should be incorporated beneath the asphalt surface layer when designing the structure of the additional pavement layer.

## Figures and Tables

**Table 1:** Performance parameters of DP4000-III multi-hammer crusher.

Maximum Drop Height	Hammer Weight	Single-Hammer Working Area
160 cm	445~500 kg	(20.32 × 2.54) cm^2^

**Table 2:** Parameters for each structural combination.

Layer	Crushing on Sub-Base	Crushing on Subgrade
Asphalt layer thickness (cm)	18	18
Modulus of asphalt layer (MPa)	1200	1200
Thickness of substrate (cm)	20	None
Modulus of substrate (MPa)	1000	None
Thickness of fractured layer (cm)	20	20
Modulus of fractured layer (MPa)	200/300/400/500/600	200/300/400/500/600
Cracked layer thickness (cm)	20	20
Cracked layer modulus (MPa)	400/600/800/1000/1200	400/600/800/1000/1200
Modulus of soil base (MPa)	60	60

## Data Availability

All data, models, and code generated or used during the study appear in the submitted article.
